# Indeterminate Cell Histiocytosis Mimicking Rosacea

**DOI:** 10.7759/cureus.12850

**Published:** 2021-01-22

**Authors:** Andrew S Fischer, Angela G Zaladonis, Paul Subrt, Jaime Tschen, Sylvia Hsu

**Affiliations:** 1 Dermatology, Elite Dermatology, Katy, USA; 2 Dermatology, Temple University Lewis Katz School of Medicine, Philadelphia, USA; 3 Dermatology, Katy Westside Dermatology, Katy, USA; 4 Dermatology, Baylor College of Medicine, Houston, USA

**Keywords:** indeterminate cell histiocytosis, rosacea, ich

## Abstract

Indeterminate cell histiocytosis (ICH) is a rare proliferative disorder of histiocytes, which display morphologic and immunophenotypic characteristics of both Langerhans cell histiocytosis (LCH) and non-Langerhans cell histiocytosis (NLCH). We describe an unusual clinical presentation of ICH mimicking rosacea and provide a relevant review of the literature.

## Introduction

Indeterminate cell histiocytosis (ICH) is a rare proliferative disorder of histiocytes, which display morphologic and immunophenotypic characteristics of both Langerhans cell histiocytosis (LCH) and non-Langerhans cell histiocytosis (NLCH). In our case, the patient presented with rosacea-like erythema and confluent papules. Immunohistochemical staining revealed histiocytes that were positive for S-100, CD1a, and CD68, and negative for Langerin. To the best of our knowledge, this is the first reported case of ICH mimicking rosacea.

## Case presentation

**Figure 1:**
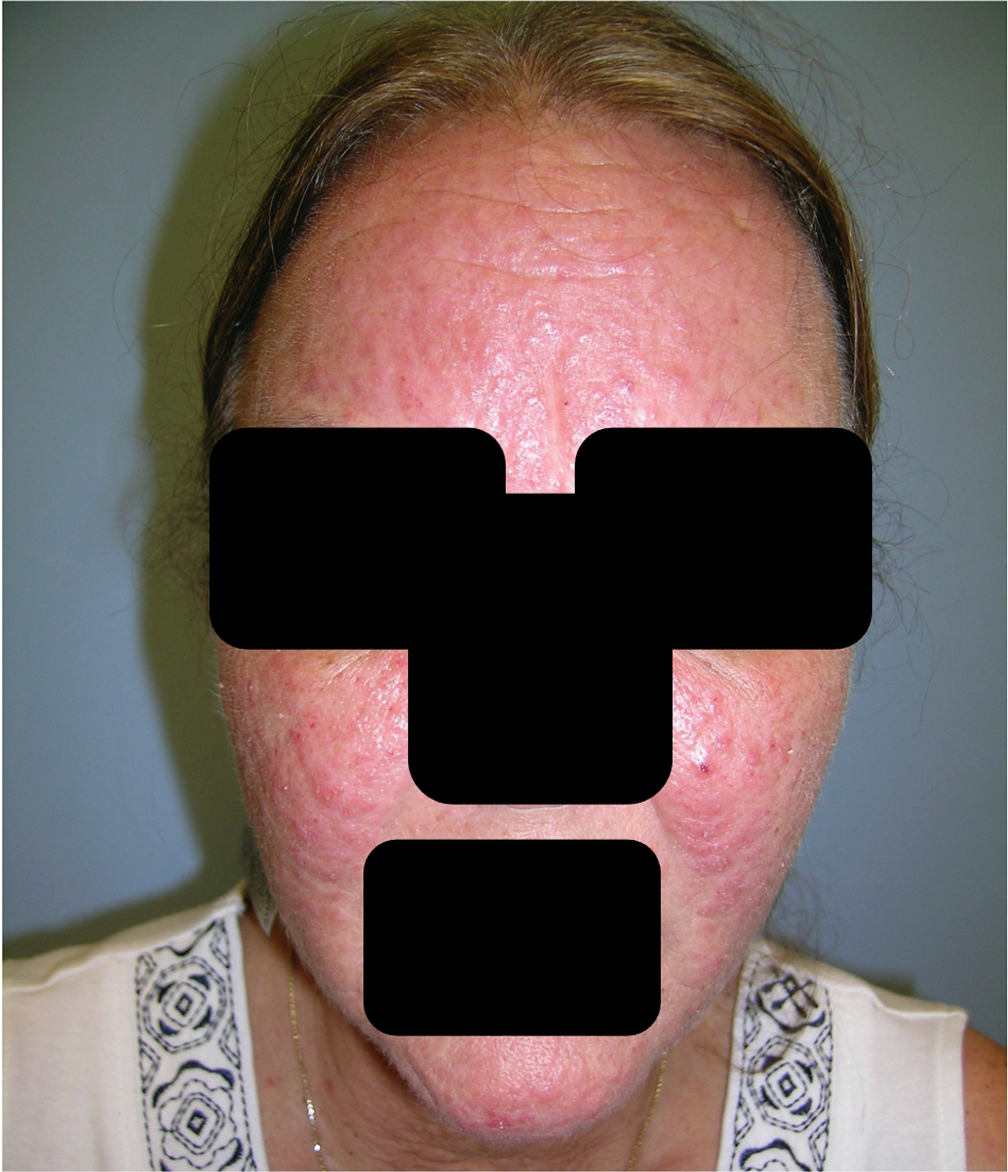
Clinical photograph of the face at the time of presentation to our clinic, showing extensive erythematous papules on the face and anterior neck.

A 55-year-old woman in otherwise good health presented with a history of burning and confluent erythematous papules on the face and arms for the preceding six months (Figure 1). Prior to being referred the patient had been treated for rosacea unsuccessfully. A biopsy was taken which suggested Langerhans cells on H&E. Additional biopsies of the chin and the arm were then performed.

**Figure 2:**
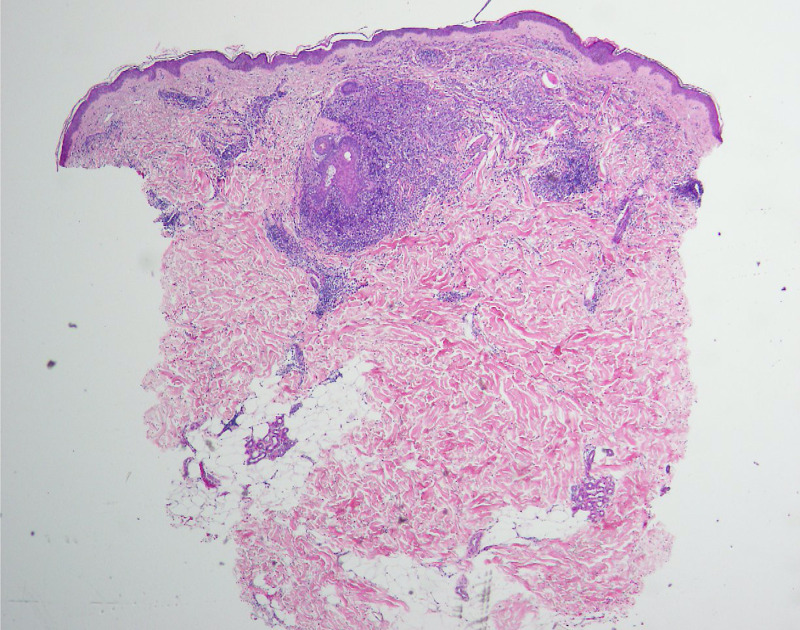
H&E photomicrograph at low power, showing a nodular infiltrate in the upper dermis.

**Figure 3:**
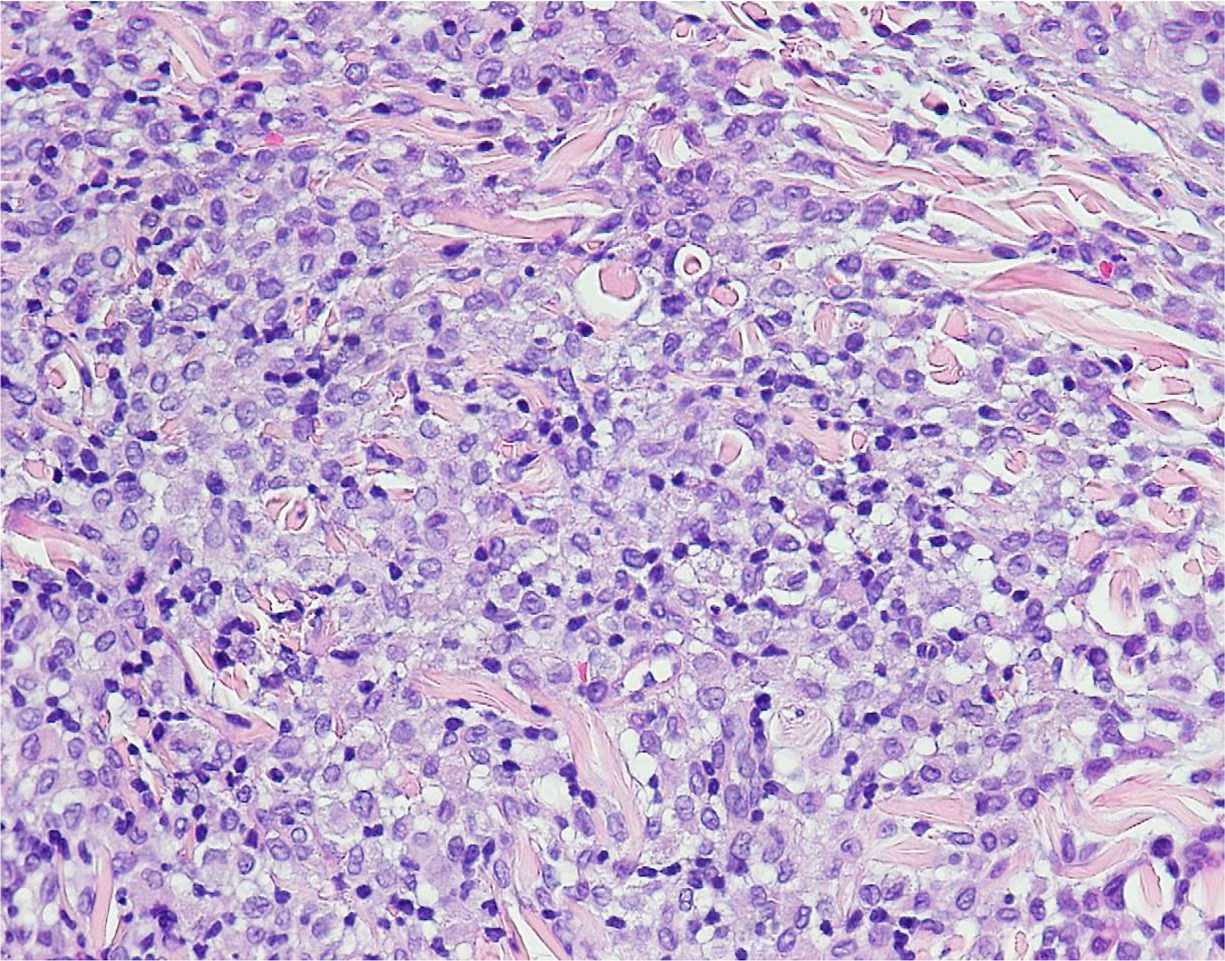
H&E photomicrograph, high power, showing mononuclear infiltrate.

**Figure 4:**
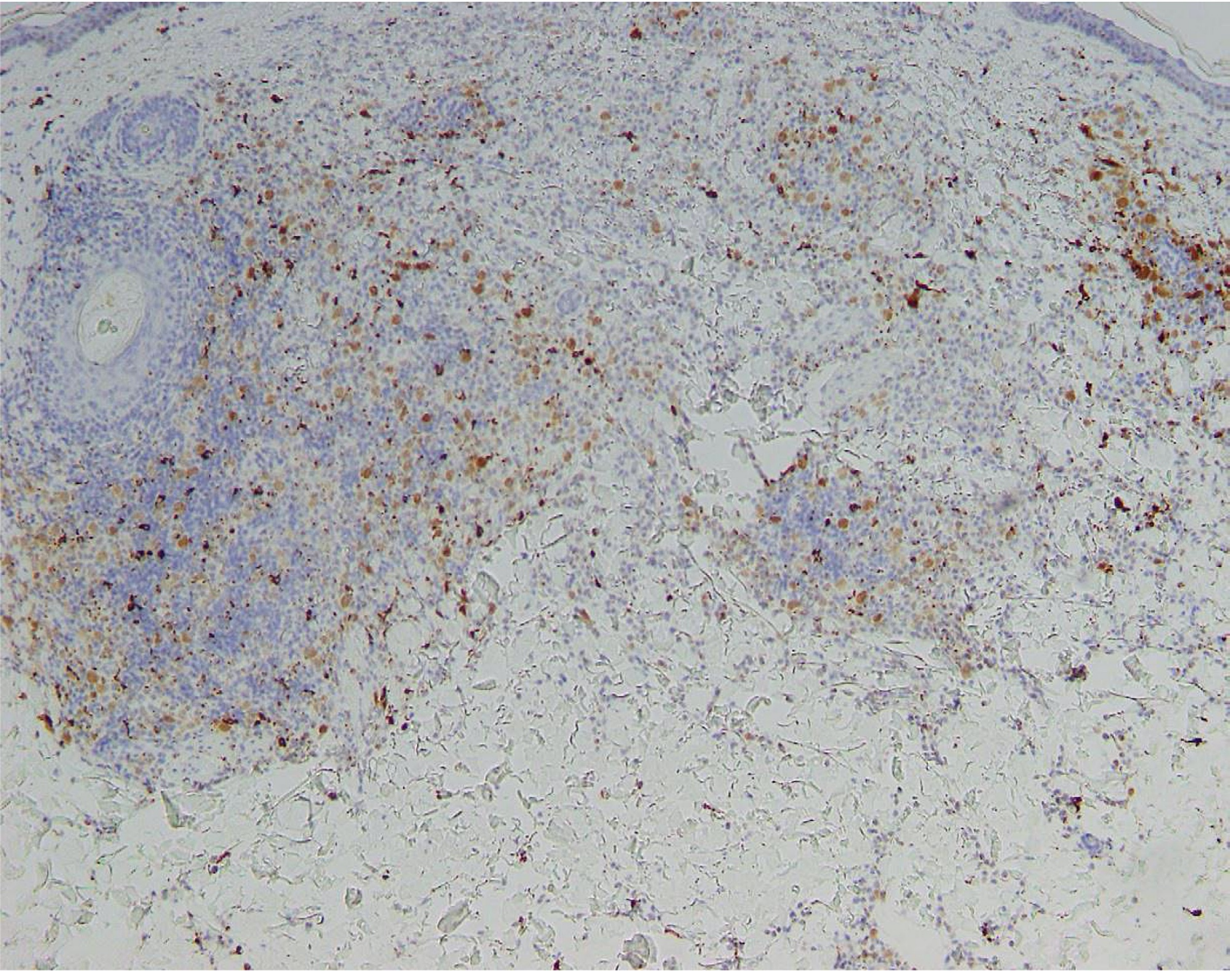
Photomicrograph of CD68 immunostain, showing scattered positivity.

**Figure 5:**
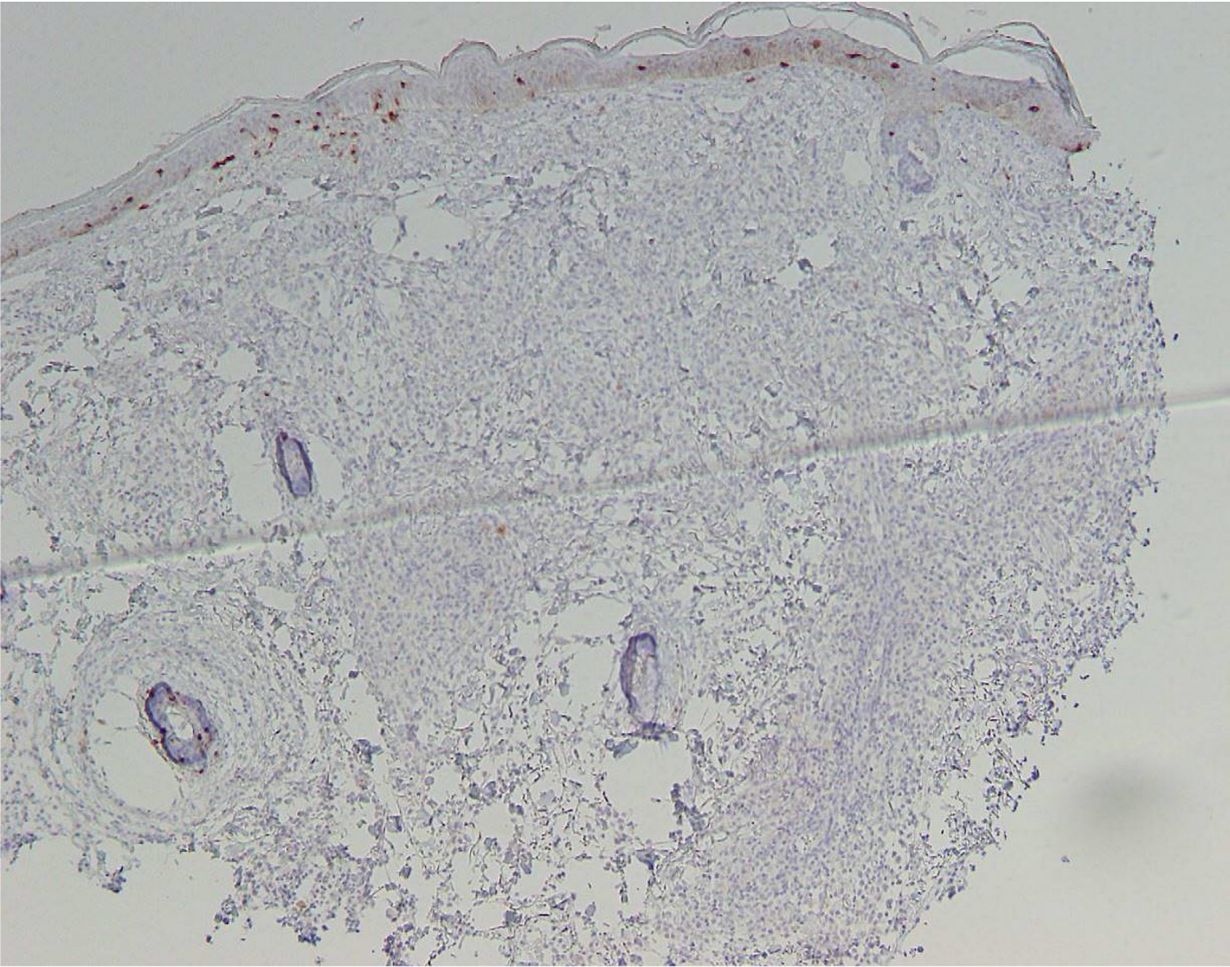
Photomicrograph of Langerin immunostain, showing lack of expression within the infiltrate.

Histopathologic examination demonstrated a dense nodular infiltrate of mononuclear cells (Figures 2-3). Immunoperoxidase staining of this infiltrate was strongly positive for CD1a and S100 (not shown), as well as less densely positive for CD68 (Figure 4). Staining for Langerin was performed and was negative (Figure 5). No appreciable neutrophils or eosinophils were present, and there was no significant epidermotropism. The presence of CD68, a monocyte-macrophage marker, in combination with the S100 and CD1a is typical of LCH; however, the lack of Langerin makes this case consistent with ICH. At this point the patient was referred to the hematology/oncology department.

**Figure 6:**
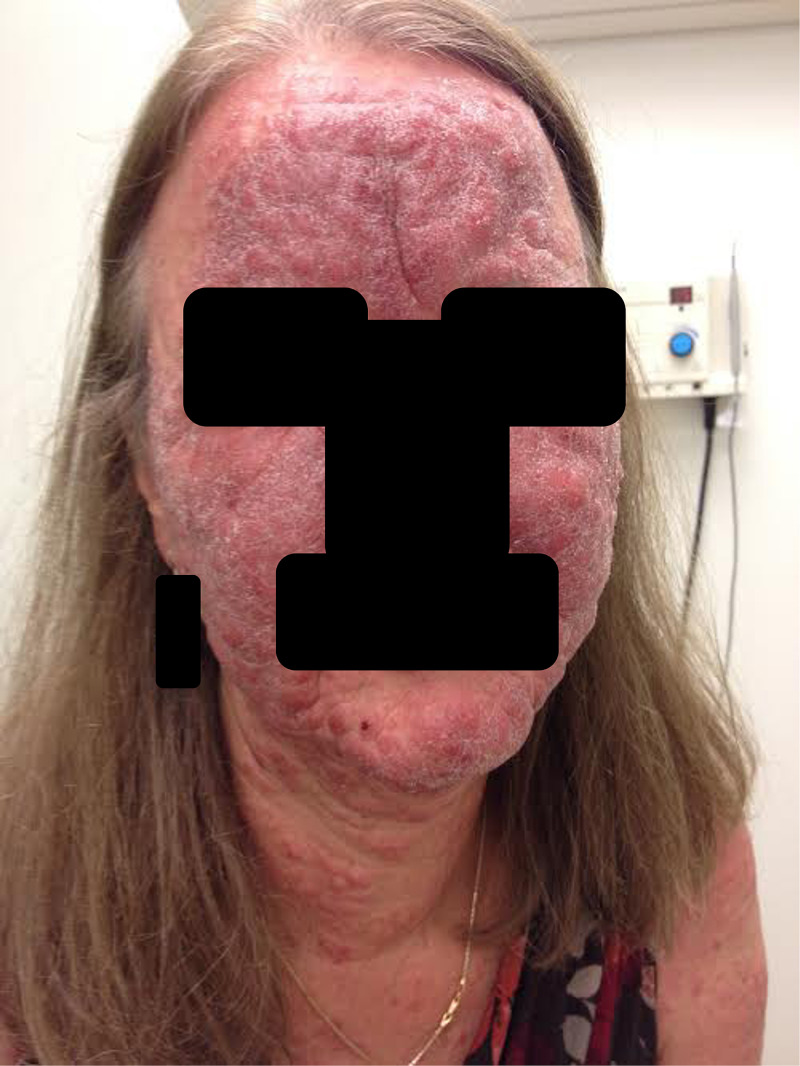
Clinical photograph of the face at six month follow-up, showing progression to thickened plaques.

**Figure 7:**
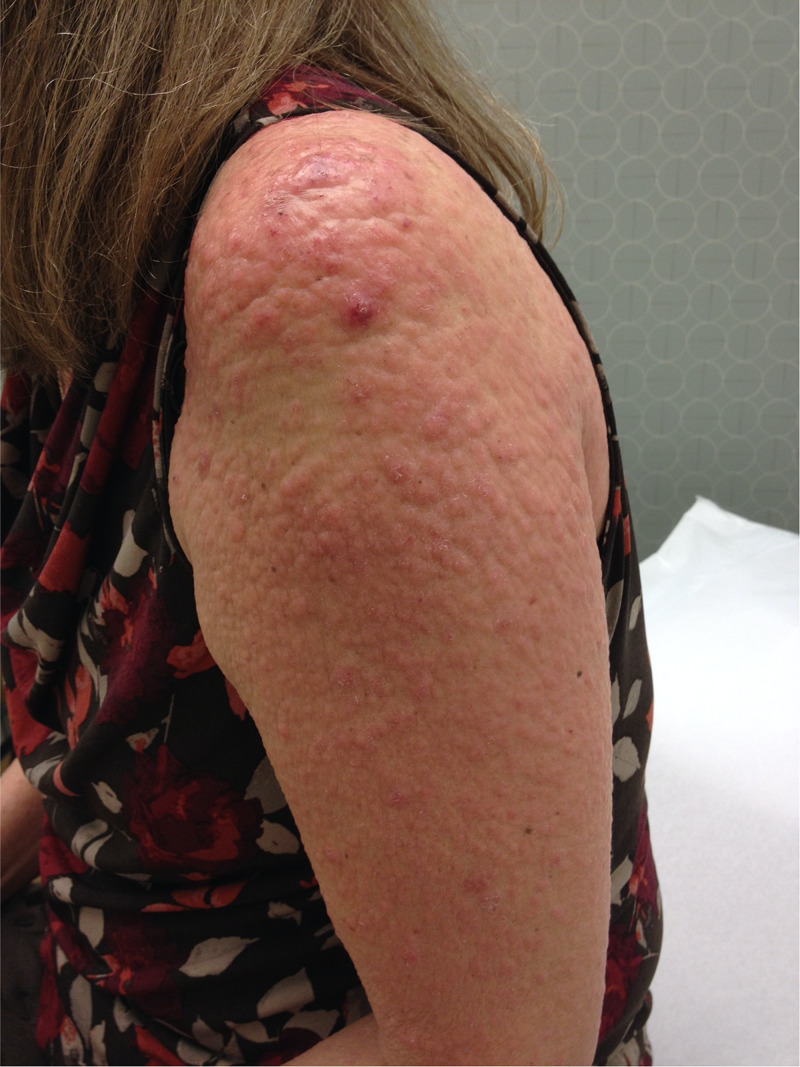
Clinical photograph of the upper arm at six month follow-up, showing progression beyond the head and neck.

At follow-up six months later the lesions had progressed into thicker, more violaceous plaques with some fine scaling (Figures 6-7). She had not yet seen a hematology/oncology consultant, but had started thalidomide in the interim and reported some subjective benefit despite overall interval worsening.

## Discussion

The histiocytoses are a heterogeneous group of disorders characterized by the proliferation of dendritic cells and cells of the monocyte-macrophage system [1]. They share a common bone marrow CD34+ progenitor cell. Traditionally, the histiocytoses have been split into those of Langerhans cells (LCH) and those of cells that derive from the monocyte-macrophage system (non-Langerhans cell histiocytosis, NLCH). Langerhans cells of LCH are immunophenotypically positive for S-100, CD1a, Langerin (CD207), and ultra-structurally contain cytoplasmic Birbeck granules. Although the Langerhans histiocytoses have traditionally been broken down into four clinical variants, there is significant overlap, suggesting a single disease with variable presentation. Unlike Langerhans cells, the cells of NLCH primarily display markers of the monocyte-macrophage system (KPI [CD68], Ki-M1p, HAM56), or of dermal dendrocytes (Factor XIIIa). They characteristically lack S100 and CD1a. Ultrastructurally, they display phagolysosomes, but lack Birbeck granules. 

The first case of ICH was reported in 1985 [2]. ICH was originally thought to be a variant of LCH as the lesional cells expressed both S100 and CD1a. However, they lacked Birbeck granules. The cells of ICH were then shown to express markers of the macrophage system KP1 (CD68), Ki-M1p, HAM56, Factor XIIIa [3-5]. This discovery brought about a shift in the categorization of ICH and it was proposed that ICH represents a distinct entity displaying features of both LCH and NLCH [5]. Most recently, it has been hypothesized that ICH may more appropriately be considered a variant of NLCH instead of an overlap between LCH and NLCH. This hypothesis is driven by the observation that most reported cases of ICH predominantly show clinical features of NLCH [1]. The routine expression of S-100 in ICH is the major drawback to the hypothesis of ICH as a NLCH-variant; however, aberrant expression of S-100 in NLCH has been reported [6].

Indeterminate cell histiocytosis is a rare disorder with less than 50 cases reported in the literature. In contrast to LCH, which has a predilection for children and adolescents, ICH has no clear predilection for sex or age, and there is one reported congenital case [5, 7]. There exist both solitary and generalized variants, which typically begin with firm, red to brown papules that may resolve spontaneously or progress and display a waxing or waning course. Ulceration may be present and lesions typically age to a yellow-brown hue. The trunk and extremities are the most common areas of involvement. However, involvement of the head and neck (with eyelids and ears) [1, 5, 8] and the genitals [1] have been reported as well. Extracutaneous and mucosal involvement is rare, but involvement of the bone [1, 9], cornea [10], and conjunctiva [1] has been reported.

Onset of most cases is spontaneous. However, some cases appear to represent a reaction to an inflammatory trigger such as scabies or pityriasis rosea [11-12]. In these cases, the lesions of ICH were distributed in the pattern of the preceding disease, suggesting an isotopic response [13]. The clinical course of ICH is variable with most patients experiencing partial or complete remission without aggressive intervention [5, 14]. Various treatments have been attempted with varying response, including phototherapy, radiation, and chemotherapy. Disease progression, visceral involvement, and death have been reported [5, 9]. ICH has been associated with malignancies: twice with low grade B-cell lymphoma [15], and once with mast cell leukemia [13], myelomonocytic leukemia [15], and monocytic leukemia [16].

Our patient presented with facial erythema and papules and with symptoms of burning and irritation that bore a striking resemblance to rosacea. To date, there have been no cases reported of ICH mimicking rosacea. There has been one case of ICH limited to the face and neck [8]. This patient lacked the conspicuous rosacea-like papules and background erythema in our patient. Rather, she presented with the typical asymptomatic, discrete red-brown papules of 1-5 mm on the face and neck, which ultimately resolved spontaneously with hyperpigmentation. Acneiform [17-18] and rosacea-like [18] cases of cutaneous Rosai-Dorfman disease have been reported. Unlike our case, the lesional cells of this NLCH stain negatively for CD1a and emperipolesis is a common finding [19].

## Conclusions

Indeterminate cell histiocytosis is a rare histiocytosis that features an overlapping immunochemical and cytologic phenotype between Langerhans and non-Langerhans histiocytoses. Our case demonstrates that ICH should be considered in the differential diagnosis of progressive, rosacea-like eruptions, which are refractory to treatment, especially when they involve less common sites for rosacea such as the extremities.
